# 2450: Factors associated with urban youth and parent perceptions of the preventability of their emergency department visit for an assault-related injury

**DOI:** 10.1017/cts.2017.278

**Published:** 2018-05-10

**Authors:** Antony Gatebe Kironji, Tina Cheng, Vanya Jones, Sarah Lindstrom Johnson, Joel Fein, Leticia Ryan

## Abstract

OBJECTIVES/SPECIFIC AIMS: To identify factors associated with urban youth and parent perceptions of the preventability (PoP) of the youth’s medically attended assault injuries in order to guide future violence prevention strategies. METHODS/STUDY POPULATION: Assault-injured youth (n=180; ages 10–15; 60% male; 96% African-American) and their parents were recruited from 2 pediatric emergency departments (EDs) in Baltimore and Philadelphia between June 2014 and June 2016. Data on demographics, circumstances of injury, injury severity, and perceptions of the injury were collected from chart review and in-person interviews with youth and parents using previously validated instruments. Within youth and parent groups, we compared those who reported “definitely true” when asked if the event that brought them to the ED could have been prevented to those who reported “maybe true” or “unlikely” using χ^2^ testing. RESULTS/ANTICIPATED RESULTS: In total, 68 (37.8%) youth and 123 parents (68.3%) reported that the injury was definitely preventable. Youth who were injured indoors [OR 2.13 (95% CI 1.17, 3.88), *p*=0.013] or considered their injury not serious [OR 4.82 (95% CI 1.78, 13.11), *p*=0.002] were more likely to perceive injury preventability and those who reported being the victim were less likely to perceive injury preventability [OR 0.26 (95% CI 0.01, 0.67), *p*=0.005]. Bullying and use of weapons were not associated with youth PoP. Parents were significantly more likely to perceive preventability when the person/people involved were known by the youth [OR 1.94 (95% CI 1.04, 3.62), *p*=0.037] and when the injury occurred indoors [OR 1.96 (95% CI 1.04, 3.69), *p*=0.038]. Similar to youth, parental report of bullying was not associated with parent PoP. Injury severity, and victim role of their child were also not associated with parent PoP. DISCUSSION/SIGNIFICANCE OF IMPACT: A prior violent injury is a major risk factor for future injuries and homicides. Through our work we were able to identify factors associated with youth and parent perception of preventability of injuries in a high risk population. Youth who felt victimized were less likely to perceive their injury as preventable. In addition, parents were more likely to perceive the injury as preventable when their injured child knew those involved in the incident. This work can inform violence prevention strategies and potentially identify opportunities to reduce intentional injuries in urban youth.

